# Contextual Anthropocentrism and Animal Welfare Attitudes Among German Livestock Farmers: Evidence from Survey Data

**DOI:** 10.3390/ani16172666

**Published:** 2026-08-25

**Authors:** Marcus Mergenthaler, Iris Schröter

**Affiliations:** Department of Agriculture, South Westphalia University of Applied Sciences, 59494 Soest, Germany

**Keywords:** animal welfare, livestock farmers, anthropocentrism, HEXACO, personality traits, positive animal welfare, biological functioning, Germany

## Abstract

Farm animal welfare research increasingly recognizes that farmers’ welfare decisions are shaped by ethical orientations. This study examines associations between livestock farmers’ contextual anthropocentrism, personality traits, farm structural characteristics, and links to animal welfare attitudes. Survey data from 619 German livestock farmers were analyzed using descriptive statistics, reliability analysis, correlations, and an ordinary least squares regression model. An anthropocentric orientation index (AOI) showed acceptable internal consistency (Cronbach’s alpha = 0.72; mean = 3.68 on a 1–5 scale). Higher emotionality, agreeableness, and openness were negatively associated with anthropocentric orientation. Organic farming, keeping of suckling cows, dairy cows, and laying hens were also negatively associated with anthropocentric orientations. The model explained a modest share of variance (R^2^ = 0.147; adjusted R^2^ = 0.119). Correlations indicated that higher anthropocentric orientations were more closely aligned with appreciation of biological functioning indicators and less aligned with positive welfare indicators, including species-typical behavior and natural outdoor access. The findings indicate tentatively that contextual anthropocentrism among livestock farmers might be operationalized empirically and might be linked to personality, farm structure, and welfare interpretation. Future research should show if contextual anthropocentrism may inform more differentiated animal welfare communication that accounts for farmers’ distinct ethical and practical animal welfare orientations.

## Simple Summary

Livestock farmers make everyday decisions that affect farm animal welfare. This study examines how German livestock farmers differ in their views on the use of animals for human purposes. Survey data from 619 livestock farmers were used to construct an anthropocentric orientation index. Farmers with higher emotionality, agreeableness, and openness expressed less anthropocentric orientations toward animals. Stronger anthropocentric orientation was linked to greater emphasis on productivity and management-compatible indicators and less emphasis on species-typical behavior and positive animal experiences. The findings suggest that animal welfare communication should account for differences among farmers, but the results are exploratory and do not show causal effects.

## 1. Introduction

With a growing understanding of animals’ consciousness [1], scientific, public, and policy concern about farm animal welfare in food production continues to increase [2]. Scientific approaches to animal welfare commonly distinguish between welfare as biological functioning and welfare as affective experience [3,4]. However, animal welfare science is systematically shaped by logistical and funding constraints and the scope of research is ‘pragmatically biased’ [5]. As livestock farmers are central actors in the practical, day-to-day realization of animal welfare [6,7], their welfare-related attitudes are embedded in economic constraints, legal standards, market incentives, professional norms, and social expectations. Their views on animal welfare therefore combine ethical beliefs about animals with practical judgements [8,9,10]. This makes livestock farming a particularly relevant context for examining how animal welfare is interpreted within human use, production systems, and economic decision-making.

A key conceptual lens for analyzing ethical orientations toward animals is anthropocentrism. Normative anthropocentrism denotes a moral position in which human interests are given priority over the interests of non-human animals [11]. In livestock farming, anthropocentric reasoning assumes context-specific forms. It may be expressed through the acceptance of animal use for food production, economic and practical welfare trade-offs, and reliance on human-defined legal standards. These orientations define the legitimacy and limits of animal welfare in relation to human needs, human-designed production systems, and institutional standards [12,13]. Previous research suggests that farmers’ ethical and animal welfare orientations are heterogeneous.

However, the individual correlates of anthropocentric orientations among livestock farmers remain insufficiently understood. Personality traits may be relevant because studies in general populations link emotionality, agreeableness, and openness to empathy, concern for animals, and meat-related ethical attitudes [14,15,16,17,18,19,20]. Agricultural applications are less common, although existing studies indicate that personality traits can be associated with farming styles, risk preferences, and participation in animal welfare or sustainability programs [21,22,23,24]. What remains unresolved is whether anthropocentric orientations among livestock farmers can be measured empirically, whether they are systematically associated with personality traits and other farmer and farm characteristics, and how they relate to animal welfare attitudes.

**Figure 1:**
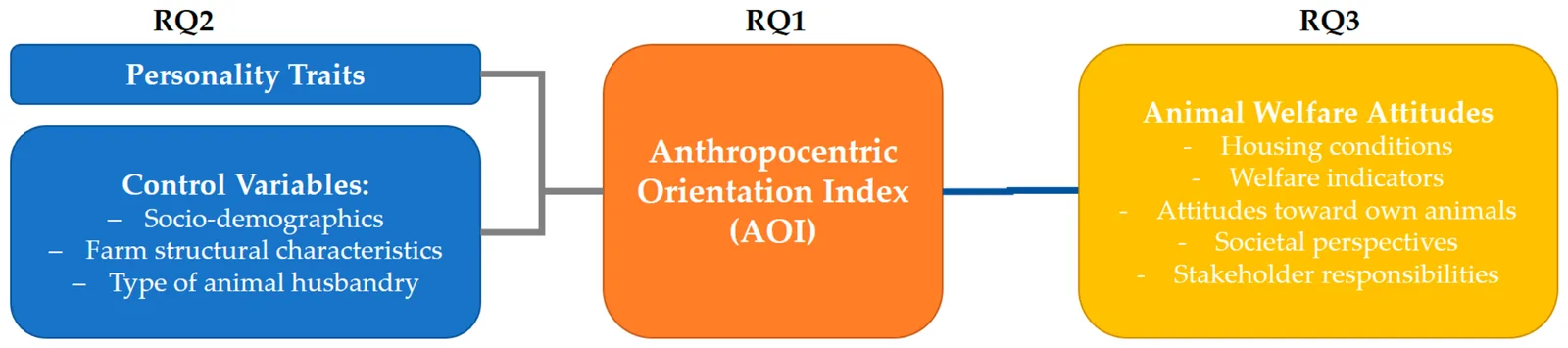
Conceptual framework linking contextual anthropocentrism to personality traits while controlling for farmer and farm characteristics as well as exploring the association with animal welfare attitudes.

Against this background, the study conceptualizes contextual anthropocentrism as an empirical construct located between ethical animal use reasoning, farmer and farm related factors, and farmers’ welfare interpretations. The research questions (RQ) therefore follow this conceptual sequence: the study first examines whether an anthropocentric orientation index (AOI) can be constructed as an internally consistent measure (RQ1), then analyzes its associations with farmer and farm characteristics with a special focus on personality traits (RQ2), and finally explores how it relates to animal welfare attitudes (RQ3). The analytical framework guiding the empirical analysis is summarized in Figure 1.

### 1.1. Research Aim

This study examines whether livestock farmers’ contextual anthropocentrism can be operationalized empirically and whether this orientation is associated with personality traits while controlling for farmer and farm characteristics and whether an anthropocentric orientation index (AOI) can be validated by animal welfare attitudes.

### 1.2. Research Questions

RQ1: To what extent can livestock-specific contextual anthropocentrism be captured as an internally consistent index?

RQ2: How is contextual anthropocentrism associated with personality traits while controlling for farmer and farm characteristics?

RQ3: How can contextual anthropocentrism be validated by farmers’ animal welfare attitudes?

## 2. Theoretical Background

Anthropocentrism provides a conceptual entry point for examining human-centered orientations toward farm animals. In environmental ethics and welfare economics, anthropocentric reasoning refers to approaches in which human interests, preferences, or welfare are treated as the primary or exclusive basis for moral and social evaluation [12,25,26,27]. Recent agricultural and resource economics research has questioned whether this human-centered framing is sufficient when sentient animals are directly affected by production and consumption decisions [28]. For livestock farming, this issue is empirically relevant because farm animals are both sentient beings and part of economically organized production systems [29]. In livestock production, such reasoning is not limited to abstract claims of human moral priority over animals, as described in normative ethics [11]. It is expressed in applied judgements about the legitimacy of animal use for food production, the prioritization of human concerns, the affordability of animal products, the acceptability of intensive husbandry conditions, and the sufficiency of legally defined welfare standards. Because minimum welfare standards develop with changing scientific and ethical understandings, legal sufficiency should also be interpreted as a human-defined and historically contingent welfare boundary [30]. This interpretation is consistent with research showing that animal welfare attitudes in farming are embedded in ethical beliefs, economic pressures, legal standards, and practical production contexts [5,6,7,8,9,31]. These dimensions define animal welfare in relation to human use, production feasibility, market affordability, and human-defined institutional standards. Thus, contextual anthropocentrism in livestock farming is a multidimensional orientation rather than a narrow measure of indifference toward animal welfare.

Animal welfare science provides the conceptual basis for interpreting how anthropocentric orientations relate to different welfare indicators. Welfare assessment is not value-free, because scientific indicators are selected and interpreted in relation to ethical concerns about animals’ quality of life [3,5,31,32]. A widely used distinction separates welfare as biological functioning from welfare as affective experience and opportunities for natural or species-typical behavior [33]. Biological functioning approaches emphasize health, normal physiology, growth, reproduction, and the prevention of disease or injury [3,4]. Positive animal welfare extends this perspective by emphasizing positive affective states, rewarding experiences, agency, competence, resilience, and quality of life beyond the mere absence of suffering [5,34,35,36]. These perspectives overlap in practice, but they direct attention to partly different welfare indicators.

Farmers’ welfare orientations are shaped by the conditions under which livestock production takes place. Animal welfare decisions are embedded in professional experience, human–animal interaction, production systems, market requirements, legal standards, advisory systems, and public expectations [6,9,37]. Prior research indicates that farmers’ attitudes toward animal welfare are heterogeneous and can differ according to values, perceived feasibility, production context, and the way welfare is measured or communicated [6,8,10,38,39]. Observable and management-relevant indicators such as health, feed intake, productivity, and handling safety may therefore be especially salient in practical farm decision-making, whereas indicators linked to positive affective experience, agency, or species-typical behavior may require broader welfare framings.

Empirical work shows that farmer attitudes and empathy can be associated with animal welfare indicators, which supports treating farmer orientations as relevant for welfare outcomes [40]. Personality traits may help explain variation in these orientations. The HEXACO model distinguishes honesty–humility, emotionality, extraversion, agreeableness, conscientiousness, and openness to experience [41,42]. The brief HEXACO inventory provides a compact measurement instrument for these six domains [43]. Research in general populations has linked personality traits, empathy, ethical concern for animals, meat consumption, and support for animal welfare legislation [14,15,16,17,18,19,20,44]. Evidence from agricultural contexts is more limited. Existing studies indicate that personality and personal values may be relevant for understanding farmers’ motivations, advisory needs, and engagement with animal welfare [22,45].

Taken together, the literature suggests that anthropocentric orientations in livestock farming should be understood as context-specific ethical and practical framings of animal use and animal welfare. The conceptual logic of the present study is summarized in Figure 1. Contextual anthropocentrism is expected to be reflected in how farmers interpret animal welfare. A stronger anthropocentric orientation does not necessarily imply rejection of animal welfare. Rather, it may be associated with a welfare framing in which health, productivity, safe handling, legal compliance, affordability, and operational feasibility receive stronger emphasis. By contrast, lower anthropocentric orientation may be more closely associated with welfare indicators related to positive affective states, naturalness, behavioral expression, and animals’ own experiential interests. The empirical analysis therefore examines three linked issues: whether anthropocentric orientation can be captured as an internally consistent index, how this index is associated with personality traits while controlling for farmer and farm characteristics, and how it relates to reported animal welfare attitudes.

## 3. Materials and Methods

To address the research questions, the study used an online survey of 619 German livestock farmers. The survey design made it possible to combine information on anthropocentric orientation, HEXACO personality traits, socio-demographic characteristics, farm structure, husbandry type, and reported animal welfare attitudes within one empirical framework.

### 3.1. Survey Design and Recruitment

The survey was conducted as part of a research project on acceptance factors of innovations in livestock farming [46] from December 2022 to January 2023. The questionnaire was developed using previous studies, project-specific expertise, and pre-testing [6,47]. The full online questionnaire was administered in German and contained 51 questions, including filter-dependent questions. The announced completion time was approximately 25 min and it finally took respondents 31.9 (±37.4) min. The questionnaire used different response formats, including single-choice questions, multiple-selection tasks, ranking tasks, slider scales, open numeric entries, and five-point Likert-type scales. Not all questionnaire items were used in the present analysis as the survey was part of a broader project. The analysis focused on variables related to farm structure, socio-demographic characteristics, HEXACO personality traits, animal welfare attitudes, responsibility attributions, and the animal ethics items used to construct the AOI. A structured overview of the questionnaire domains used in this study is provided in Table 1.

Participants were recruited through three pathways. The informal pathway used social media and messenger services (*n* = 155). The formal pathway used professional networks, newsletters, and invitations in farming magazines (*n* = 158). The commercial pathway used quota sampling by a market research service provider (*n* = 306). Only respondents in the commercial pathway received financial compensation of EUR 25. The other respondents participated with intrinsic motivation without payment.

The sample is not representative of all German livestock farmers. It combines self-selected respondents and commercially recruited participants. Recruitment pathway may therefore be associated with respondent characteristics, response quality, animal welfare attitudes, and motivation to participate. This is especially relevant because only commercially recruited respondents received financial compensation. The recruitment pathways and possible implications are discussed in more detail by Mergenthaler et al. [48]. As indicated by the cross-sectional design, findings should be interpreted as associations within this sample and not as population estimates for German livestock farming.

### 3.2. Measures

The questionnaire contained predominantly closed questions on farm structure, socio-demographic characteristics, personality traits, animal welfare attitudes, and anthropocentric orientation. Farm structural variables included animal husbandry type, organic farming, and farm size. Farm size was reported in hectares. For the regression model, farm size was entered in 100 ha units to improve coefficient readability. Socio-demographic variables included age, gender, and education.

Personality traits were measured using the 24-item brief HEXACO inventory [43]. The six traits were honesty–humility, emotionality, extraversion, agreeableness, conscientiousness, and openness to experience. Respondents rated the personality items on a five-point Likert scale ranging from 1 = strongly disagree to 5 = strongly agree. The resulting trait scores were treated as quasi-metric variables in the analyses.

Animal welfare attitudes were measured using several item blocks. Respondents selected the three most important housing conditions for animal well-being and the three most important indicators used to determine the well-being of farm animals. Additional Likert-scale items assessed attitudes toward animal husbandry, animal welfare, safe handling, digitalization, and socio-political aspects. Respondents also rated the perceived obligation of different societal groups to ensure animal welfare.

### 3.3. Construction of the Anthropocentric Orientation Index (AOI)

Animal ethics attitudes were measured using six items designed to operationalize contextual anthropocentrism in livestock farming. The theoretical premise was that anthropocentrism in livestock production is not expressed only as an abstract claim of human moral priority over animals. It also becomes visible in applied judgements about animal use, human consumption, affordability, legal sufficiency, and the acceptability of production-related welfare trade-offs. This understanding is consistent with anthropocentrism as a human-centered moral and evaluative orientation [11,12,26,27] and with research showing that animal welfare attitudes in farming are embedded in economic pressures, legal standards, production contexts, and practical feasibility [7,8,9,31]. Table 2 summarizes how each of the six items represents a specific dimension of contextualized anthropocentrism in livestock farming.

The six items were selected because they refer directly to livestock-specific forms of anthropocentric reasoning. They capture agreement with animal use for human consumption, human entitlement to use animals, the prioritization of human problems over animal welfare concerns, affordability-based justification of intensive production conditions, and reliance on legal minimum standards as an acceptable welfare boundary. The AOI therefore focuses on contextual anthropocentrism within agricultural animal production rather than on general attitudes toward all human uses of animals.

Each item was measured on a five-point Likert scale, with higher values indicating stronger agreement with the respective anthropocentric statement. The AOI was constructed as an unweighted mean index for conceptual and empirical reasons. Conceptually, the six items were treated as related manifestations of a broader livestock-specific anthropocentric orientation rather than as separate subscales. Empirically, the number of items was too small to support a robust multidimensional scale structure, and the reliability analysis indicated acceptable internal consistency for exploratory research. Equal weighting was used because there was no theoretical or empirical basis for assigning stronger weights to individual dimensions. The resulting AOI should therefore be interpreted as an exploratory composite measure, not as a fully validated psychometric scale.

### 3.4. Statistical Analysis

The statistical analysis followed the exploratory aim of the study and combined descriptive statistics, reliability analysis, bivariate association measures, and multivariable ordinary least squares (OLS) regression. Descriptive statistics were used to summarize the sample, the AOI items, the aggregate AOI, personality traits, socio-demographic characteristics, farm structural variables, husbandry types, and animal-welfare-related attitudes. For metric or quasi-metric variables, means and standard deviations were reported. For categorical or binary variables, frequencies and percentages were used. The tables combine descriptive statistics with association measures in order to keep the empirical presentation compact. To improve readability, the tables use consistent rounding, and significance markers.

The internal consistency of the AOI was assessed using a reliability analysis and Cronbach’s alpha. In addition to the overall alpha coefficient, item-level diagnostics were examined including inter-item correlations, corrected item-total correlations, and Cronbach’s alpha if each item was deleted. These diagnostics were used to assess whether the six items could be combined into one exploratory composite index of livestock-specific contextual anthropocentric orientation. Because all items used the same five-point response scale and there was no theoretical or empirical basis for differential weighting, the AOI was calculated as the unweighted mean of the six items, with higher values indicating stronger anthropocentric orientation.

Bivariate associations between the AOI and animal-welfare-related variables were analyzed according to the measurement level of the respective variables. Point biserial correlations were used for binary variables derived from the selection tasks, including selected housing conditions and selected welfare indicators. Spearman correlations were used for Likert-scale items on animal welfare attitudes and perceived obligations of societal actors. These correlations were used to examine whether anthropocentric orientation was associated with different animal welfare attitudes, including biological functioning, management-relevant, positive welfare, naturalness-oriented, and responsibility-related dimensions.

To examine adjusted associations between farmers’ personality traits and contextual anthropocentrism, an ordinary least squares (OLS) regression model was estimated with the AOI as the dependent variable. OLS regression was chosen because the AOI was constructed as a mean index and treated as a quasi-metric outcome. The independent variables of substantive interest were the six HEXACO personality traits: honesty–humility, emotionality, extraversion, agreeableness, conscientiousness, and openness to experience. The model additionally controlled for socio-demographic and farm structural characteristics. Age was included as a metric variable measured in decades. Farm size was included as a metric variable, measured in units of 100 hectares. University education, female gender, and organic farming were included as dummy variables. Husbandry type was represented by dummy variables, with sow keeping used as the reference category. The resulting coefficients therefore describe adjusted differences in AOI scores relative to the respective reference groups or, for metric variables, adjusted changes in AOI scores associated with a one-unit increase in the predictor.

OLS results were reported using unstandardized regression coefficients (B), standardized regression coefficients (Beta), significance levels, model fit statistics, and explained variance. The coefficient of determination (R^2^) and adjusted R^2^ were used to assess the share of variance in AOI explained by the model. The F-test was used to assess the overall statistical significance of the regression model. Because the study is exploratory, *p*-values were interpreted as indicators of statistical association within the sample rather than as confirmatory hypothesis tests. Model diagnostics were inspected for the final OLS specification. Multicollinearity was assessed using tolerance and variance inflation factor (VIF) values. Residual distribution was examined using standardized residuals, a histogram of standardized residuals, a normal P–P plot, and a scatterplot of standardized residuals against standardized predicted values. The results are interpreted as exploratory associations because of the non-representative sample, the cross-sectional design, and the modest explanatory power of the model. All analyses were conducted using IBM SPSS Statistics, Version 27.

## 4. Results

**Figure 2:**
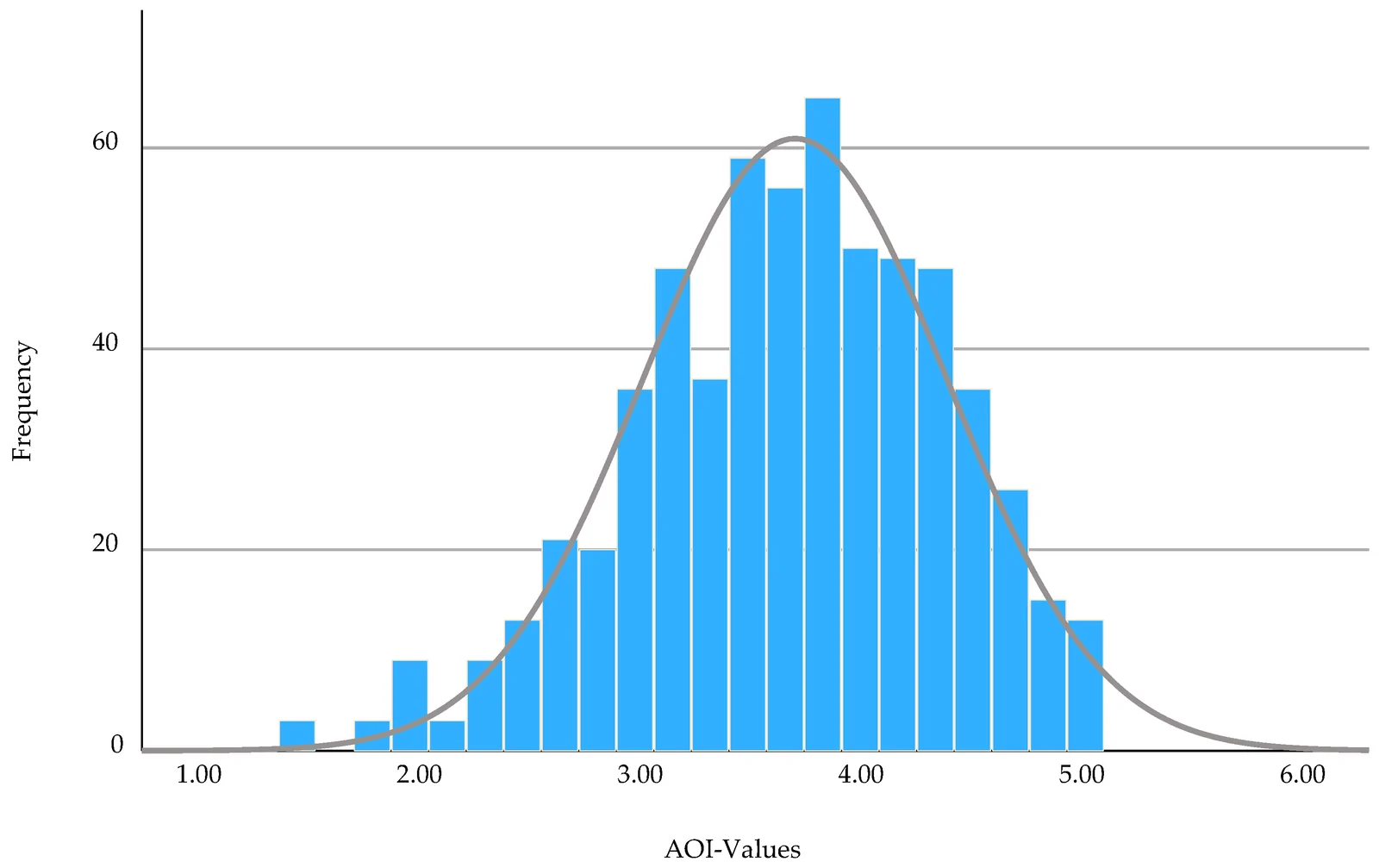
Histogram of the anthropocentric orientation index (AOI) on a scale from 1 to 5.

In livestock farming, contextual anthropocentrism was operationalized through acceptance of human use, production-oriented welfare trade-offs, affordability arguments, and legal sufficiency reasoning. Table 3 and Figure 2 summarize the AOI and its constituent items. Internal consistency of the six AOI items was assessed using Cronbach’s alpha. The six items showed acceptable internal consistency for exploratory research (Cronbach’s alpha = 0.717; standardized alpha = 0.720). All inter-item correlations were positive and ranged from 0.192 to 0.382, with a mean inter-item correlation of 0.300, indicating that the items were related but not redundant. Corrected item total correlations ranged from 0.382 to 0.517. Cronbach’s alpha if item deleted ranged from 0.656 to 0.698. No item was removed. The AOI was consequently constructed as an unweighted mean index. The highest item mean was observed for agreement that it is completely fine that animals are kept for human consumption. The lowest item mean was observed for the statement that cheap meat, eggs, and dairy products justify keeping farm animals under intensive conditions.

The distribution of the AOI in Figure 2 shows variation across respondents. The AOI had a mean of 3.68, a median of 3.67, and a standard deviation of 0.69, with values ranging from 1.5 to 5.0. The distribution was slightly left-skewed (skewness = −0.402) and close to mesokurtic (kurtosis = −0.063), indicating moderate concentration in the upper-middle part of the scale without strong deviation from symmetry. This pattern suggests that contextual anthropocentrism was common in the sample, but not uniform.

Table 4 presents descriptive statistics and OLS regression results in one table. The interpretation focuses primarily on the direction, size, and statistical significance of the regression coefficients, while the descriptive columns provide information on the distribution of the predictors in the estimation sample. The model explained 14.7% of the variance in the AOI (R^2^ = 0.147; adjusted R^2^ = 0.119). The overall model was statistically significant (F = 5.346; *p* < 0.001), with a standard error of the estimate of 0.647. The explanatory power is modest and should be interpreted accordingly. Diagnostic checks did not indicate severe multicollinearity. Tolerance values ranged from 0.410 to 0.950, and VIF values ranged from 1.052 to 2.439. Standardized residuals ranged from −3.504 to 2.673 and a standard deviation of 0.984. Inspection of the residual histogram, normal P–P plot, and residual-versus-predicted scatterplot did not indicate a pattern that would invalidate the exploratory OLS interpretation.

Among the HEXACO traits, emotionality, agreeableness, and openness were negatively associated with the AOI. This means that respondents with higher scores on these traits tended to show lower contextual anthropocentrism, after accounting for the other variables in the model. Honesty–humility, extraversion, and conscientiousness were not statistically significant. The pattern suggests that contextual anthropocentrism is more closely related to traits associated with emotional sensitivity, interpersonal orientation, and openness to broader perspectives than to the other personality dimensions included in the model.

Socio-demographic control variables showed no clear association with the AOI. Age, gender, and university education were not statistically significant in the reported model. Farm size was also not significantly associated with anthropocentric orientation. By contrast, organic farming was negatively associated with the AOI, indicating lower contextual anthropocentrism among organic farms compared with conventional farms, after adjustment for personality traits, socio-demographic characteristics, farm size, and husbandry type.

For husbandry type, sow keeping served as the reference category. Compared with this reference group, dairy cow farming, suckling cow farming, and laying hen farming were associated with lower AOI scores. Pig fattening and cattle fattening showed weaker negative associations, while the remaining husbandry categories were not statistically significant. These husbandry-type differences suggest that contextual anthropocentrism varies across production contexts. However, because some husbandry categories are comparatively small, the corresponding coefficients should be interpreted as exploratory adjusted differences rather than stable population estimates.

Overall, the regression results indicate that contextual anthropocentrism was more clearly associated with selected personality traits, organic farming, and husbandry type than with socio-demographic characteristics or farm size. The pattern supports the interpretation of the AOI as an empirically observable farmer orientation that varies across individual dispositions and production contexts. At the same time, the modest model fit and cross-sectional design require a cautious, non-causal interpretation.

After identifying farm and farmer characteristics associated with the AOI, the following analyses examine how contextual anthropocentrism is reflected in different animal welfare attitudes. Table 5, Table 6 and Table 7 report bivariate associations between the AOI and animal-welfare-related attitudes. The tables are structured so that descriptive information is presented first, followed by the corresponding association with the AOI. For binary variables, point biserial correlations are reported. For Likert-type and slider-scale variables, Spearman correlations are used.

Table 5 reports frequencies for selected housing conditions and welfare indicators together with point biserial correlations. Conditions related to biological functioning, such as good air quality and access to high-quality feed and water, were selected frequently and showed positive correlations with the AOI. Sufficient space was also selected frequently. It was negatively correlated with the AOI. Items more closely linked to positive animal welfare, such as outdoor run with natural ground cover and species-typical behavior, showed negative correlations with the AOI.

The second part of Table 5 shows that indicators such as appearance of the animal, good state of health, and feed intake behavior were frequently selected as welfare indicators. These indicators did not all show the same association with the AOI. Animal performance and feed intake behavior were positively correlated with the AOI, whereas species-typical behavior, atmosphere in the barn, and positive state of mind were negatively correlated. This pattern is consistent with a stronger orientation toward visible, functional, or productivity-related indicators among respondents with higher AOI scores.

Table 6 reports correlations between the AOI and Likert-scale measured attitudes. Several general welfare-related statements were positively correlated with the index, including pride in animal performance, the perceived importance of welfare on the farm, safe handling of animals, and digitalization as conducive to greater animal welfare. These results indicate that higher AOI scores do not imply a general rejection of animal welfare. Instead, they suggest that stronger contextual anthropocentrism is associated with a functional, managerial, and production-compatible conception of animal welfare.

Additional statements in Table 6 point to links between the AOI and perceived structural pressures. Agreement that livestock farmers need planning security, that livestock farming has no future in Germany, and that legislative changes are insufficiently communicated was positively correlated with the AOI. Agreement that livestock farms should become more accessible to the public and that livestock farming is too focused on the experiences of previous generations was negatively correlated with the index. These associations suggest that the index is also connected with broader views on the social and regulatory context of livestock farming.

Table 7 reports perceived obligations of societal groups to ensure animal welfare. Farmers attributed the highest obligation to farmers themselves, followed by consumers, retail, processing companies, and politics/government, while animal welfare organizations received the lowest mean obligation score. The AOI was negatively correlated with perceived obligations for most groups, especially politics/government and animal welfare organizations. This suggests that lower AOI scores were associated with assigning greater animal welfare responsibility to several actor groups, including farmers, politics/government, retail, processing companies, and animal welfare organizations.

## 5. Discussion

This study examined associations between livestock farmers’ anthropocentric orientation index (AOI), personality and animal welfare orientations. For RQ1, the AOI captured a livestock-specific orientation that combines normative anthropocentrism, economic pragmatism, acceptance of legal minimum standards, and functional welfare reasoning. For RQ2, AOI values were associated with selected HEXACO traits. Higher AOI scores were associated with lower emotionality, agreeableness, and openness. For RQ3, higher AOI scores were not linked to a simple rejection of animal welfare, but to a specific anthropocentric welfare framing: welfare is primarily interpreted through visible, functional, and management-compatible indicators. Indicators associated with positive welfare and affective state receive comparatively less emphasis. The AOI is also associated with attitudes related to animal welfare governance. Given the modest explanatory power of the regression model and the cross-sectional design, these interpretations should be understood as exploratory and hypothesis-generating. The associations do not allow conclusions about causal mechanisms.

The findings contribute to debates on animal welfare governance and farmers’ moral reasoning by showing that contextual anthropocentrism is not adequately understood as simple indifference toward animal welfare. Rather, it reflects a welfare framing in which human use, economic feasibility, legal sufficiency, and production system constraints shape which indicators are regarded as legitimate and practically relevant. This creates a governance tension: animal welfare transformation depends on broader animal sentience perspectives, but such perspectives may be contested when they appear insufficiently compatible with farm-level autonomy, planning security, and economic viability.

The six items that constitute the AOI share a common orientation toward human use, production conditions, and the prioritization of human, economic, or legal concerns in relation to farm animal welfare. The acceptable internal consistency of the index supports its use as an exploratory measure. The AOI captures related dimensions of livestock-specific contextual anthropocentrism, including human priority claims, instrumental animal use reasoning, affordability-based trade-offs, and legal sufficiency reasoning. However, high scores should not be interpreted as direct evidence of moral disregard for animals per se.

Compared with an earlier, independent sample from 2018 of German livestock farmers [23], the present sample displayed a very similar HEXACO personality profile, suggesting substantial descriptive stability across the two independently recruited farmer samples. Compared with the general population samples [23], both farmer samples show a stable profile of higher honesty–humility, higher conscientiousness, and lower emotionality. Differences in extraversion, agreeableness, and openness are smaller, inconsistent across comparison samples, or sensitive to the measurement instrument. The negative associations in the present study between emotionality, agreeableness, openness, and the AOI are consistent with research linking these traits to empathy, ethical concern, and attitudes toward animals in non-farmer populations [14,15,16,17,18,19,20]. The regression design does not establish that personality traits cause attitudes, but it indicates systematic associations.

The associations with socio-demographic and farm structural control variables require cautious interpretation. Organic farming was negatively associated with AOI. This may reflect differences in production standards, farmer self-selection, normative system orientation, or market context. Keeping of suckling cows, dairy cows, and laying hens were also negatively associated with the AOI relative to sow keeping. These differences may reflect variation in husbandry systems, market contexts, system inherent human–animal interaction, or the normative framing of species-specific welfare. The present data cannot separate these mechanisms.

The correlations with animal welfare attitudes further suggest that higher AOI scores were more closely linked to biological functioning and management-relevant welfare indicators than to indicators of positive animal welfare. This does not imply disregard for animal welfare. Rather, welfare appears to be interpreted through an anthropocentric lens in which health, productivity, safe handling, and operational feasibility receive stronger emphasis than species-typical behavior, natural outdoor access, and positive affective states [5,49]. This supports the relevance of distinguishing biological functioning from positive welfare when analyzing farmers’ welfare orientations [3,36].

The attitudinal associations also suggest that contextual anthropocentrism may be linked to farmers’ perceived position under external transformation pressure. Higher AOI scores may indicate professional boundary setting, where welfare demands are evaluated in relation to autonomy, operational control, entrepreneurial decision-making, and bureaucratic burden. This interpretation is consistent with research showing that farmers’ welfare attitudes are embedded in economic pressures, professional norms, practical feasibility, and ambivalent societal expectations [7,8,9]. It also reflects a tension within stewardship: farmers may understand themselves as responsible animal caretakers, while defining good welfare primarily through human judgement, farm-level feasibility, and managerial control. This ethical tension remains constitutional for animal husbandry because animals are sentient beings with their own experiential interests, including pain, fear, comfort, positive affect, agency, and opportunities for meaningful behavioral engagement [1,5,32,34,36].

Responsibility attributions extend this interpretation from individual attitudes to animal welfare governance. Stronger AOI scores were associated with narrower obligation attributions to all surveyed societal actors. In light of the foundational ethical tensions of animal husbandry, this might indicate dissolving or strategic externalization of responsibility. Within the limits of the cross-sectional design, it may especially indicate skepticism toward externally imposed welfare governance, particularly when public, political, or civil society claims are perceived as insufficiently sensitive to farm practice, and market constraints [10,37,50]. Because farm animals have subjective experiences of their own lives, welfare governance should also address affective states, behavioral opportunities, and positive experiences within the production system [35,36,49]. At the same time, the pattern may also reflect constrained agency and partly reflect a defensive attribution pattern: farmers may regard themselves as willing or responsible in principle, while locating the practical limits of welfare improvements outside their own immediate sphere of action [9]. This ambiguity is important because a purely stewardship-based interpretation would remain incomplete if it treated farmer responsibility as sufficient by itself. The present data cannot determine whether such attributions express justified recognition of structural constraints, strategic externalization of responsibility, or both. The results therefore suggest that animal welfare governance must address how welfare standards are justified, which indicators are considered legitimate, and how responsibility is distributed among farmers, markets, consumers, retailers, processors, policy actors, and civil society organizations.

The association between contextual anthropocentrism, perceived external pressure, and narrower responsibility attribution may partly reflect personality patterns that distinguish livestock farmers from general population samples. One possible interpretation is that farmers with lower emotionality may be less receptive to welfare claims framed around animals’ subjective experiences, agency, or interests independent of human use. However, the present data cannot determine whether this association reflects stable personality-related differences, occupational selection, professional socialization, production system experience, or responses to external criticism. These findings suggest, cautiously, that animal welfare transformation may benefit from learning and advisory formats that support reflection on animal behavior, affective states, and animals’ perspectives. This implication remains tentative and subject to future research because the study did not measure learning processes, advisory participation, or actual welfare outcomes. Such development should not take the form of paternalistic intervention by external actors who define farmers as morally deficient. It should instead be embedded in participatory and practice-based settings in which farmers retain agency and contribute their own experiential knowledge. Direct observation of positive and negative affective states, structured reflection on animal behavior, peer-to-peer learning, and jointly developed welfare assessments with societal actors may help connect animals’ experiential interests with farmers’ existing stewardship responsibilities. The aim would be to expand, rather than replace, farmers’ welfare frameworks by linking affective experience, behavioral agency, and animal-centered considerations with economic feasibility and professional autonomy.

## 6. Limitations

This study has several limitations. First, the sample is non-representative. It combines informal, formal, and commercial recruitment pathways and is therefore subject to selection bias. Respondents may differ from the broader population of German livestock farmers in motivation, interest in animal welfare issues, farm structure, response quality, or willingness to participate in online surveys. In addition, the differential use of financial compensation across recruitment pathways may have contributed to differences in respondent motivation, response quality, and animal-welfare-related attitudes. These potential pathway effects cannot be fully separated in the present exploratory analysis. They have been discussed by Mergenthaler et al. [48] in more depth. Therefore, our results should not be interpreted as representative population estimates.

Second, the study relies on self-reported attitudes. Such measures are useful for analyzing perceptions and values, but they do not necessarily reflect actual on-farm behavior or animal welfare outcomes. Social desirability may have affected responses, particularly for items related to animal welfare, legal standards, and responsibility attributions. The survey did not include objective animal welfare indicators, observational data, or external validation of reported farm practices.

Third, the explanatory power of the regression model is modest. The model explains a statistically significant but limited share of variance in the AOI. Some relevant factors may not have been included in the model, including perceived economic pressure, market conditions, production contracts, advisory exposure, regional context, regulatory experience, and farm profitability. The reported associations should therefore be interpreted as partial and exploratory.

Fourth, the AOI is understood as a multidimensional measure of livestock-specific contextual anthropocentrism. The items capture different dimensions of anthropocentric reasoning, including human use, affordability-based trade-offs, intensive production conditions, and legal sufficiency. This breadth is conceptually complex for livestock farming, where animal welfare is negotiated within production systems and human-defined standards. The index combines different contextual dimensions of anthropocentric orientations and treats these related dimensions as one composite measure. Future research should examine its dimensional structure through confirmatory scale development procedures and compare it with established measures of animal attitudes, empathy, and moral concern for animals.

Fifth, the cross-sectional design does not support causal inference. The associations between personality traits, farming system, husbandry type, and anthropocentric orientation may reflect selection processes, socialization, family farm history, production system norms, or unobserved confounders. Reverse or reciprocal relationships cannot be excluded. Longitudinal or mixed-method designs would be needed to examine how these attitudes develop over time.

## 7. Conclusions

This study indicates that livestock-specific contextual anthropocentrism can be cautiously operationalized as an exploratory empirical construct. The AOI captures shared orientations concerning the legitimacy of animal use. Higher AOI scores were associated with lower emotionality, agreeableness, and openness to experience. The explanatory power of the regression model remained modest, however, and the findings should be interpreted as associations within a non-representative sample rather than as causal relationships or population estimates.

The findings refine the interpretation of farmer heterogeneity in animal welfare research. Differences between livestock farmers should not be understood only as differences in support for, or resistance to, animal welfare. They also reflect different assumptions about what animal welfare means, which welfare indicators are considered legitimate, which trade-offs are accepted, and who has authority to define welfare standards. Stronger contextual anthropocentrism appears to combine human-centered animal use reasoning, economic pragmatism, legal sufficiency reasoning, and professional boundary setting. It may also reflect concerns that animal welfare transformation could reduce entrepreneurial autonomy, increase bureaucratic burdens, or constrain farm-level decision-making. The AOI therefore captures a tension between welfare defined through human judgment, managerial feasibility, and institutional standards and a broader animal-centered perspective that gives independent weight to animals’ subjective experiences, behavioral opportunities, and experiential interests. Personality traits were associated with how farmers positioned themselves within this tension, but the findings do not support deterministic or causal interpretations. The results do not show whether these associations reflect stable personality-related differences, occupational selection, professional socialization, production system experience, responses to societal criticism, or unobserved confounders.

Given the exploratory, non-representative, and cross-sectional design, the practical implications should be read as practice-oriented hypotheses rather than tested intervention effects. Animal welfare transformation should neither treat farmers as a homogeneous group nor frame particular personality profiles as moral deficiencies. Participatory and practice-based approaches may be more appropriate than paternalistic interventions. Farmer-led observation of affective states and behavior, peer-to-peer learning, structured reflection, and jointly developed welfare assessments could help connect animal-centered considerations with farmers’ existing stewardship responsibilities. The objective would be to expand, rather than replace, farmers’ welfare frameworks by linking positive welfare, behavioral agency, and animals’ experiential interests with animal health, economic feasibility, planning security, and professional autonomy.

The AOI remains an exploratory measure and requires further validation. Future research should examine its dimensional structure and relationship with established measures of empathy, animal attitudes, perceived economic pressure, professional identity, and regulatory experience. Representative and longitudinal studies are needed to clarify whether anthropocentric orientations arise primarily from individual dispositions, occupational selection, professional socialization, production system conditions, or interactions among these factors. Combining farmer-reported attitudes with observational and animal-based welfare indicators would further show whether different ethical orientations are associated with measurable differences in welfare practices and outcomes.

## Figures and Tables

**Table 1:** Overview of questionnaire domains used in the present analysis.

Questionnaire Domain	Main Content	Response Format	Role in the Present Analysis
Farm and husbandry characteristics	Husbandry type, organic/conventional farming, farm size.	Single-choice and numeric entries.	Control variables in OLS regression.
Socio-demographics	Age, gender, education.	Single-choice and numeric entries.	Control variables in OLS regression.
Personality traits	24-item brief HEXACO inventory.	Five-point Likert-type scale.	Key explanatory variables in OLS regression.
Animal ethics items	Six anthropocentric orientation items.	Five-point Likert-type scale.	Construction of AOI; reliability analysis; dependent variable in OLS regression.
Animal welfare attitudes	Housing conditions, welfare indicators, attitudes toward own animals and livestock farming, perceived obligation of societal actors to ensure animal welfare.	Multiple-selection tasks, ranking task, Likert-type scales, slider scale.	Bivariate correlations with AOI.

**Table 2:** Conceptual justification of the six AOI items as dimensions of contextual anthropocentrism in livestock farming.

AOI Item	Dimension of Contextualized Anthropocentrism	Conceptual Justification
I think people who are against keeping animals for meat production are too sentimental.	Delegitimization of animal-centered moral concern.	This item captures an anthropocentric evaluation of opposition to meat production as excessive emotionality. It frames moral concern for animals as sentimentality and thereby gives priority to the legitimacy of animal use for human food production.
It is completely fine that animals are kept for human consumption.	Acceptance of animal use for human consumption.	This item captures a basic anthropocentric premise of livestock farming: animals may legitimately be kept for human consumption. It places the justification of animal keeping in human nutritional, cultural, and market purposes.
Humans fundamentally have the right to use animals as they see fit.	Explicit human use entitlement.	This item represents the most direct normative expression of anthropocentrism. It states a fundamental human entitlement to use animals and therefore gives human purposes priority in the human–animal relationship.
Nowadays, there is too much fuss about animal welfare, when there are so many problems regarding humans that need to be solved.	Priority of human problems over animal welfare.	This item captures an explicit hierarchy between human and animal concerns. It operationalizes anthropocentrism as the prioritization of human social, economic, or political problems over animal welfare claims.
The production of cheap meat, eggs, and dairy products justifies keeping farm animals under intensive conditions.	Affordability-based welfare trade-off.	This item captures an applied production–economic form of anthropocentrism. It treats intensive husbandry conditions as acceptable when they support affordable animal products for humans, thereby subordinating animal welfare ambitions to human economic benefit and food affordability.
The agricultural keeping of livestock according to legal minimum standards corresponds to my personal ideal.	Legal sufficiency as human-defined welfare boundary.	This item captures reliance on human-defined legal minimum standards as sufficient for acceptable livestock keeping. It reflects contextualized anthropocentrism because the benchmark for adequate welfare is set by institutional regulation rather than by broader animal-centered criteria.

**Table 3:** Single items and the aggregate anthropocentric orientation index (AOI), with descriptive statistics and Cronbach’s alpha for the index and Cronbach’s alpha if the respective item is deleted.

	Mean	SD	Min	Max	Cronbach’s Alpha (If Item Is Deleted)
**Aggregate index: anthropocentric orientation index (AOI)**	**3.68**	**0.69**	**1.5**	**5.0**	**0.72**
I think people who are against keeping animals for meat production are too sentimental	4.05	1.07	1.0	5.0	0.70
It is completely fine that animals are kept for human consumption	4.60	0.73	1.0	5.0	0.70
Humans fundamentally have the right to use animals as they see fit	3.36	1.18	1.0	5.0	0.66
Nowadays, there is too much fuss about animal welfare, when there are so many problems regarding humans that need to be solved	3.70	1.08	1.0	5.0	0.67
The production of cheap meat, eggs, and dairy products justifies keeping farm animals under intensive conditions	3.12	1.24	1.0	5.0	0.68
The agricultural keeping of livestock according to legal minimum standards corresponds to my personal ideal	3.22	1.07	1.0	5.0	0.66

**Table 4:** Descriptive statistics and the OLS results of personality traits, socio-demographics, farm structural characteristics and types of animal husbandry used in the regression analyses of the anthropocentric orientation index (AOI).

	Descriptive Statistics		OLS-Regression			
	Mean	SD	B-Coef	Beta-Coef	*p*-Value	Sig.
constant	-	-	4.281	-	<0.001	***
**HEXACO-personality traits**						
- Emotionality	2.730	0.612	−0.130	−0.115	0.005	**
- Openness	3.270	0.606	−0.102	−0.089	0.028	*
- Agreeableness	3.060	0.502	−0.114	−0.084	0.033	*
- Extraversion	3.683	0.529	0.040	0.031	0.443	
- Honesty-humility	4.218	0.543	0.063	0.050	0.226	
- Conscientiousness	3.618	0.567	0.074	0.061	0.132	
**Socio-demographic characteristics**						
- Female (dummy)	0.120	-	−0.123	−0.058	0.160	
- University education (dummy)	0.310	-	−0.069	−0.046	0.256	
- Age (decades)	4.796	1.181	0.004	0.007	0.853	
**Farm structural characteristics**						
- Organic (dummy)	0.100	-	−0.244	−0.108	0.009	**
- Farm size (100 ha)	1.840	3.710	0.010	0.055	0.159	
**Type of animal husbandry**						
- sow keeping (reference)	0.139	-	-		-	
- suckling cows (dummy)	0.094	-	−0.650	−0.300	<0.001	***
- dairy cows (dummy)	0.268	-	−0.212	−0.140	0.018	*
- pig fattening (dummy)	0.282	-	−0.164	−0.104	0.072	+
- laying hens (dummy)	0.038	-	−0.289	−0.089	0.047	*
- cattle fattening (dummy)	0.122	-	−0.185	−0.085	0.087	+
- turkey fattening (dummy)	0.017	-	−0.145	−0.028	0.490	
- poultry fattening (dummy)	0.024	-	−0.088	−0.019	0.644	
- piglet rearing (dummy)	0.014	-	0.061	0.007	0.854	

Notes: Dependent variable: anthropocentric orientation index (AOI). OLS = ordinary least squares. B = unstandardized coefficient; Beta = standardized coefficient. + *p* < 0.10, * *p* < 0.05, ** *p* < 0.01, *** *p* < 0.001.

**Table 5:** Frequencies of different animal-welfare-related aspects and their point biserial correlations with AOI.

	Relative Frequency	Point-Biserial Correlation	*p*-Value	Sig.
**In your opinion, what are the three most important housing conditions for an animal to do really well? (dummy)**				
- outdoor run with natural ground cover	0.103	−0.269	<0.001	***
- sufficient space	0.599	−0.128	0.001	**
- animal-friendly stable structure	0.267	−0.109	0.007	**
- free access to paved outdoor run	0.047	−0.104	0.010	**
- good lighting conditions	0.184	0.014	0.727	
- opportunities for activity	0.126	0.045	0.268	
- appropriate floor in the stable	0.121	0.065	0.108	
- separation options for special groups	0.095	0.097	0.016	*
- free access to good quality feed and water	0.822	0.107	0.008	**
- good air quality	0.612	0.233	<0.001	***
**In your opinion, what are the three most important indicators to determine the well-being of your animals? (dummy)**				
- exercise of species-typical behavior	0.194	−0.170	<0.001	***
- positive state of mind	0.118	−0.129	0.001	**
- atmosphere in the barn	0.239	−0.110	0.006	**
- animal group dynamics	0.074	−0.055	0.168	
- behavior towards people	0.097	−0.041	0.304	
- good state of health	0.716	−0.006	0.877	
- appearance of the animal	0.696	0.006	0.891	
- feed intake behavior	0.483	0.119	0.003	**
- animal performance	0.372	0.263	<0.001	***

Notes: AOI = anthropocentric orientation index. Point biserial correlations are reported for binary selection variables. * *p* < 0.05, ** *p* < 0.01, *** *p* < 0.001.

**Table 6:** Means and standard deviations of different animal-welfare-related attitudes and their correlation with AOI.

	Mean	SD	Correlation	*p*-Value	Sig.
**Below are some statements about your attitudes towards your own animal husbandry. Please indicate the extent to which you agree with the following statements on a scale from 1 to 5:**					
- The welfare of the animals on my farm is very important to me.	4.840	0.478	0.140	<0.001	***
- Digitalization on my farm is conducive to greater animal welfare.	3.318	1.205	0.141	<0.001	***
- I am proud of the performance of the animals on my farm.	4.387	0.787	0.222	<0.001	***
- For my own protection, safe handling of the animals on my farm is important, even if this affects their behavior.	3.670	1.086	0.354	<0.001	***
**Below you will see statements made by farmers on a different occasion. Please indicate on a scale from 1 to 5 to what extent you agree with these statements according to your own convictions.**					
- When it comes to livestock farming, livestock farmers are too focused on the experiences of previous generations.	2.434	0.959	−0.233	<0.001	***
- Livestock farms must become more accessible to the public.	3.078	1.074	−0.222	<0.001	***
- Broad social support is crucial for future-proof livestock farming.	4.203	0.856	−0.105	0.009	**
- Farm animal husbandry has no future in Germany.	2.793	1.221	0.150	<0.001	***
- Legislative changes relating to livestock farming in accordance with the legal minimum standard are primarily orientated towards societal requirements.	3.883	1.007	0.164	<0.001	***
- Changes in legislation are not sufficiently communicated with livestock farmers.	4.450	0.759	0.278	<0.001	***
- Livestock farmers need planning security in order to be able to keep farm animals.	4.843	0.455	0.281	<0.001	***

Notes: AOI = anthropocentric orientation index. Spearman correlations are reported for Likert-scale variables. ** *p* < 0.01, *** *p* < 0.001.

**Table 7:** Means and standard deviations of different societal groups’ obligation to ensure animal welfare and their correlation with AOI.

	Mean	SD	Correlation	*p*-Value	Sig.
**The obligation to ensure animal welfare in farm animals is an obligation for society as a whole. Many sections of the population can contribute to animal welfare in different ways. In your opinion, how large is the obligation of the following groups to this cause? Analogue scale from 1 to 100**					
- Animal welfare organizations	23.528	26.815	−0.329	<0.001	***
- Politics/government	66.599	28.513	−0.228	<0.001	***
- Retail	72.399	27.674	−0.135	<0.001	***
- Farmers	86.596	16.892	−0.116	0.004	**
- Processing companies	71.036	25.005	−0.098	0.015	*
- Consumers	72.748	27.014	−0.077	0.055	+

Notes: AOI = anthropocentric orientation index. Spearman correlations are reported for analogue-scale variables. + *p* < 0.10, * *p* < 0.05, ** *p* < 0.01, *** *p* < 0.001.

## Data Availability

The data supporting the findings of this study are not publicly available due to data protection regulations and privacy restrictions. The survey data contain information from livestock farmers that may allow indirect identification when combined with farm structural and socio-demographic variables. Public data sharing is therefore not possible under the applicable data protection requirements and the conditions under which informed consent was obtained.
